# 25(OH)D Serum Level in Non-Diabetic and Type II Diabetic Patients: A Cross-Sectional Study

**DOI:** 10.7759/cureus.8910

**Published:** 2020-06-29

**Authors:** Mohmmed A AlHewishel, Mohammed Bahgat, Abdullah Al Huwaiyshil, Mustafa A Alsubie, Abdullah Alhassan

**Affiliations:** 1 Internal Medicine, King Faisal University, Al-Ahsa, SAU; 2 Biomedical Sciences, King Faisal University, Al-Ahsa, SAU

**Keywords:** diabetes type ii, non-diabetic, 25 hydroxyvitamin d

## Abstract

Background

Diabetes mellitus is a major disease worldwide. In Saudi Arabia, it is considered to be the most common disease in the country. Diabetes mellitus has been also found to be associated with 25(OH)D (vitamin D) deficiency. In Saudi Arabia, sunlight is considered a major source for vitamin D. Saudi Arabia is popular for sunny weather most of the year, in which people can get vitamin D from the sun. However, vitamin D deficiency is common in Saudi Arabia, and its deficiency can increase blood glucose levels. We conducted a study to determine the reason for vitamin D deficiency in Saudi Arabia and to assess the relationship of diabetes mellitus with vitamin D.

Aim of the work

This study is aimed to assess the incidence of vitamin D deficiency in non-diabetic and type II diabetic patients in the King Faisal University (KFU) Health Center in the Al-Ahsa region.

Methods

Our study is a cross-sectional study that was carried out at the KFU Health Center in Saudi Arabia. Ethical approval was obtained from the Ethics and Research Committee at the College of Medicine at King Faisal University. The study period was from January 2016 to April 2016. We collected each patient's vitamin D serum level, glycosylated hemoglobin (HbA1c), and fasting blood glucose at the same time for each patient's particular visit to the hospital. Data were analyzed using the Statistical Package for Social Sciences (SPSS) (IBM SPSS Statistics, Armonk, NY).

Results

Our results showed that 89.53% of the patients had a vitamin D level below the normal range. There was a higher incidence of vitamin D deficiency in females (81.67%) than in males (65.27%) (p-value = 0.001). The incidence of vitamin D deficiency was greater in Saudi (82.19%) than non-Saudi patients (68.40%) (p-value = 0.001), as well as in diabetics (89.68%) than non-diabetics (76.12%) patients (p-value = 0.001). Within each group, the incidence of vitamin D deficiency was higher in females than in males. The incidence of vitamin D deficiency was highest in the age group of 21 to 40 years old (86.19%) and lowest in the age group of one to 20 years old (66.1%). The results showed an inverse relationship between the vitamin D level and both fasting blood glucose and HbA1c (independent sample t-test) were used for correlation. The mean fasting glucose was higher in the deficiency group (165.55) as compared to the insufficiency group (118.67). Also, the mean HbA1c was higher in the deficiency group (8.06) as compared to the insufficiency group (7.23) (p-value = 0.030).

Conclusions

There was a high incidence of vitamin D deficiency among KFU Health Center patients. The vitamin D level was inversely proportional to the level of fasting glucose and HbA1c. There is an evident role of vitamin D deficiency on glucose tolerance in diabetic patients.

## Introduction

Diabetes mellitus (DM) is a syndrome of chronic hyperglycemia due to relative insulin deficiency, insulin resistance, or both [1]. In 2019, the International Diabetes Federation estimated the number of people with diabetes worldwide to be nearly 463 million. Also, the number of diabetes mellitus patients is increasing worldwide and is expected to reach 578 million by 2030 [2]. 

Vitamin D is a group of steroid hormones that are mainly formed in the skin under the effect of the sun's ultraviolet B rays and then modified in the liver and kidneys to convert it to the active form (1,25-dihy­droxyvitamin D3). It is also present in food as vitamin D3 (animal source) and vitamin D2 (plant source) [3].

The active form of vitamin D functions by binding to intracellular vitamin D receptors (VDRs) which modulate gene expression. It is now known that every cell within the body has a VDR. VDRs are also present in pancreatic β-cells and vitamin D is essential for normal insulin secretion [4]. Islet cell insulin secretion is reduced in vitamin D-deficient animals and can be corrected by vitamin D supplementation [4-5]. It is well-known that obesity and a sedentary lifestyle predispose to diabetes [6-8].

A meta-analysis of 21 prospective studies showed an inverse and significant association between circulating 25(OH) vitamin D levels and the risk of type 2 diabetes [9]. Higher 25(OH) vitamin D levels were always associated with lower diabetes risk. Each 4 ng/ml increment in 25(OH)D levels was associated with a 4% lower risk of type 2 diabetes Other studies provided prospective evidence that low levels of vitamin D also predict hyperinsulinemia. It has been suggested that vitamin D may act to prevent type 2 diabetes by decreasing insulin resistance [10]. Moreover, Dalgård et al. reported an inverse association between HbA1c and 25(OH) D3 in the elderly and concluded that a high vitamin D status protects against type 2 diabetes in younger subjects to subjects older than 70 years [11].

The National Health and Nutrition Examination Survey (NHANES) III examined research in the period between 1988 and 1994 and found high evidence of an inverse association between low levels of 25(OH) vitamin D and diabetes prevalence [12]. Low vitamin D levels have also been shown to be predictive of developing type 2 diabetes in the future. 

It has been concluded that low vitamin D levels may play a significant role in the pathogenesis of type 2 diabetes. The NHANES community (2003- 2006) has assessed 9,773 U.S. adults > 18 years of age and showed a strong link between serum vitamin D levels, glucose homeosta­sis, and the evolution of diabetes. Kositsawat et al. concluded that patients with elevated hemoglobin A1c levels should be evalu­ated for vitamin D insufficiency [13].

Kumar et al. reported a positive effect of vitamin D on beta-cell function and glucose tolerance [14]. Nigil Haroon et al. conducted a systematic review of 17 randomized control trials and seven longitudinal studies that had been followed for a minimum period of one month [15]. The result with follow-up in less than three months found that vitamin D supplementation had a positive effect on glycemic control and metabolic parameters, such as insulin resistance and beta-cell dysfunction.

The Nurses' Health Study, an observational study on 83,779 females followed for more than 20 years, showed an association of the risk of type 2 diabetes increasing with low vitamin D serum levels [16]. A combined daily intake of more than 800 IU of vitamin D and 1,000 mg of calcium decreased the risk of developing type 2 diabetes by 33%. Another study showed that increasing vitamin D serum levels to normal range led to a 55% relative reduction in the risk of developing type 2 diabetes [17]. Islet cell insulin secretion is reduced in vitamin D deficient animals and can be corrected by vitamin D supplementation [4-5].

The European Diabetes Centers Study of Complications in Patients with Insulin-Dependent Diabetes Mellitus (EURODIAB) study, a case-control study, showed a reduced risk of developing type 1 diabetes by vitamin D supplementation during the first year of life [18].

In 2013, the International Diabetes Federation (IDF) Global Diabetes Scorecard shows that Diabetes Raw prevalence in the world ranges from 1.55% - 20.22%. Compared to other areas of the world, Saudi Arabia had the highest prevalence reach (20.22%) [19]. Also, vitamin D deficiency was highly prevalent in Saudi medical students [20]. Al-Elq recorded vitamin D deficiency among medical Saudi students with a very high incidence among male (92.64%) and female (99.03%) medical Saudi students [21]. In a recent study on pregnant Saudi women, Al-Faris reported vitamin D deficiency in 50% and insufficiency in 43.8% of the study sample (total of 93.8% pregnant women had a level lower than normal) [22].

This study was conducted to assess the incidence of vitamin D deficiency in non-diabetic and type II diabetic patients at the King Faisal University (KFU) Health Center in the Al-Ahsa region. We will also assess the relationship between vitamin D and glucose tolerance indicators (fasting blood glucose and hemoglobin A1c).

## Materials and methods

Research design

Our study was a cross-sectional study done at the KFU Health Center in Saudi Arabia. The study was conducted during the period from January 2016 to April 2016.

Procedure

In our research, we collected the patients' data from the KFU Health Center, after permission from the College of Medicine higher authorities and KFU polyclinic administration. Ethical approval was obtained from the Ethics and Research Committee in the College of Medicine, King Faisal University. We collected patients' data, including gender, age, nationality, and blood analysis (vitamin D, fasting blood glucose, and HbA1c levels).

Research population

Eight hundred and sixty patients were included in our study who were one year or older, non-diabetic, and type II diabetic patients and were medically free from hypertension, dyslipidemia, and were not taking vitamin D supplements. These patients were classified into subgroups of non-diabetics and diabetics according to fasting glucose and HbA1c levels.

Materials

In the KFU Health Center, vitamin D level (ng/ml) was measured by VITROS® 350 chemistry analyzer (Ortho Clinical Diagnostics, Buckinghamshire, UK). The cut-off levels of vitamin D level are divided into the following ranges: normal 50 - 70 ng/ml, insufficient 30 - 49 ng/ml, and deficient < 30 ng/ml. The fasting blood glucose was measured by Reflotron® Plus chemistry analyzer (Roche Diagnostics, Basel, Switzerland). The Hb1Ac (mg/dl) was measured by Infopia® Clover A1c analyzer (Anyangcheondong-ro, Dongan-Gu, Anyang, Gyeonggi, Korea).

Statistical analyses

The data were analyzed using the Statistical Package for Social Sciences (SPSS), version 21 (IBM SPSS Statistics, Armonk, NY). The Chi-square and independent-sample t-tests were applied, when appropriate, for comparisons between groups to determine significance.

## Results

Our results showed that out of the 860 patients, a total of 770 (89.53%) patients had vitamin D levels below normal (675 (78.49%) had a vitamin D deficiency and 95 (11.05%) had a vitamin D insufficiency). Only 90 (10.47%) out of the 860 patients had normal vitamin D levels. Six hundred and thirty-five (91.63%) females and 135 (80.84%) males had abnormal vitamin D levels (below normal level). There was a higher incidence of vitamin D deficiency in 566 females (81.67%) than in 109 males (65.27%) (Chi-square test, p-value = 0.001) (Table 1).

**Table 1:** Distribution of Different Vitamin D Levels (Normal, Abnormal, Insufficiency, and Deficiency) in Male and Female Patients

Vitamin D levels	Male		Female		Total	
	No	%	No	%	No	%
Normal (50 - 70 ng/ml)	32	19.16	58	8.37	90	10.47
Abnormal (any level below normal)	135	80.84	635	91.63	770	89.53
Insufficiency (30 - 49 ng/ml)	26	15.57	69	9.96	95	11.05
Deficiency (< 30 ng/ml)	109	65.27	566	81.67	675	78.49
Total	167	100.00	693	100.00	860	100.00

The incidence of abnormal vitamin D (below normal level) and vitamin D deficiency (< 30 ng/ml) was greater in Saudi (91.41% and 82.19%, respectively) than non-Saudi (84.42% and 68.40%, respectively) patients and the normal level was higher in non-Saudi (15.58%) than Saudi patients (8.59%). Within each nationality, the incidence of abnormal vitamin D levels and vitamin D deficiency was higher in females than in males (Chi-square test, p-value = 0.001) (Table 2).

**Table 2:** Distribution of Vitamin D Levels in Saudi and Non-Saudi Patients

Vitamin D levels	Saudi						Non-Saudi					
	Male		Female		Total		Male		Female		Total	
	No	%	No	%	No	%	No	%	No	%	No	%
Normal (50 - 70 ng/ml)	18	16.67	36	6.91	54	8.59	14	23.73	22	12.79	36	15.58
Abnormal (any level below normal)	90	83.34	485	93.09	575	91.41	45	76.27	150	87.21	195	84.42
Insufficiency (30 - 49 ng/ml)	13	12.04	45	8.64	58	9.22	13	22.03	24	13.95	37	16.02
Deficiency (< 30 ng/ml)	77	71.30	440	84.45	517	82.19	32	54.24	126	73.26	158	68.40
Total	108	100.00	521	100.00	629	100.00	59	100.00	172	100.00	231	100.00

The incidence of abnormal vitamin D levels and vitamin D deficiency was greater in diabetic (87.27% and 76.12%, respectively) than non-diabetic patients (97.62% and 89.68%, respectively), and the normal level was higher in non-diabetic (12.73%) than diabetic patients (2.38%). Within each group, the incidence of abnormal vitamin D levels and vitamin D deficiency was higher in females than in males (Chi-square test, p-value = 0.001) (Table 3).

**Table 3:** Distribution of Vitamin D Levels in Non-Diabetic and Diabetic Patients

Vitamin D levels	Non-diabetics						Diabetics					
	Male		Female		Total		Male		Female		Total	
	No	%	No	%	No	%	No	%	No	%	No	%
Normal (50 - 70 ng/ml)	41	25.79	56	9.29	97	12.73	1	5.56	2	1.85	3	2.38
Abnormal (any level below normal)	118	74.21	547	90.71	665	87.27	17	94.44	106	98.15	123	97.62
Insufficiency (30 - 49 ng/ml)	24	15.09	61	10.12	85	11.15	2	11.11	8	7.41	10	7.94
Deficiency (< 30 ng/ml)	94	59.12	486	80.60	580	76.12	15	83.33	98	90.74	113	89.68
Total	159	100.00	603	100.00	762	100.00	18	100.00	108	100.00	126	100.00

According to age, the incidence of vitamin D deficiency was highest in the age group from 21 - 40 years (86.19%) and lowest in the group from 1 - 20 years (66.1%) (Chi-square test, p-value = 0.001) (Table 4).

**Table 4:** Distribution of Vitamin D Levels According to Age Subgroups

Age in years	1 - 20		21 - 40		> 41		Total	
Vitamin D levels	No.	%	No.	%	No.	%	No.	%
Normal (50 - 70 ng/ml)	53	18.15	18	5.41	19	8.09	90	10.47
Abnormal (any level below normal)	239	81.85	315	94.59	216	91.91	770	89.53
Insufficiency (30 - 49 ng/ml)	46	15.75	28	8.41	21	8.94	95	11.05
Deficiency (< 30 ng/ml)	193	66.1	287	86.19	195	82.98	675	78.49
Total	292	100.00	333	100.00	235	100.00	860	100.00

Table 5 shows the distribution of different vitamin D in all subgroups (each was classified by gender, nationality, and presence or absence of diabetes).

**Table 5:** Distribution of Vitamin D Levels in All Subgroups Classified by Gender, Nationality, and Presence or Absence of Diabetes

Vitamin D level	Non- diabetic								Diabetic							
	Saudi				Non-Saudi				Saudi				Non-Saudi			
	Male		Female		male		female		Male		female		male		female	
	N	%	N	%	N	%	N	%	N	%	N	%	N	%	N	%
Normal (50 - 70 ng/ml)	17	18.28	35	7.94	14	25.00	21	14.58	1	6.67	1	1.25	0	0	1	3.57
Insufficient (30 - 49 ng/ml)	11	11.83	38	8.62	13	23.21	23	15.97	2	13.33	7	8.75	0	0	1	3.57
Deficient (< 30 ng/ml)	65	69.89	368	83.45	29	51.79	100	69.44	12	80	72	90	3	100	26	92.86
Total	93	100	441	100	56	100	144	100	15	100	80	100	3	100	28	100

The results showed an inverse relationship between vitamin D levels with the fasting blood glucose and HbA1c levels. The mean fasting blood was higher in the deficiency group (165.55) as compared to the insufficiency group (118.67). Also, the mean HbA1c was higher in the deficiency group (8.06) as compared to the insufficiency group (7.23). Independent-samples t-test was used for correlation (p-value = 0.030) (Table 6).

**Table 6:** Correlation Between FBG (a) and HbA1c (b) Levels in Vitamin D Deficient and Insufficient Diabetic Patients There is an inverse proportion between vitamin D levels and fasting blood glucose (FBG) and HbA1c levels.

Vitamin D levels	FBG level (a)	HbA1c level (b)
Insufficiency (30 - 49 ng/ml)	165.55	8.06
Deficiency (< 30 ng/ml)	118.67	7.23

## Discussion

Our study revealed a very high incidence of vitamin D deficiency in the patients studied but the incidence varied between the subgroups. A higher incidence of vitamin D deficiency was found in females as compared to males, in Saudi as compared to non-Saudi patients, and in diabetic as compared to non-diabetic patients. A higher incidence of vitamin D deficiency in females than males was found in Saudi and non-Saudi and in diabetic and non-diabetic patients. These results confirm previous studies done in different countries [11-12]. Many studies reported a high association between vitamin D deficiency and diabetes and suggested that vitamin D supplementation can reduce the incidence of diabetes [23-24].

The higher incidence of vitamin D deficiency in Saudi as compared to the non-Saudi patients (p-value = 0.001) can be explained by the variation in garments where the Saudi people wear clothes to limit their exposure to the sun. In a similar manner, we can explain a higher incidence of vitamin D deficiency in females as compared to males. In our community, the females wear long robes and head coverings for religious reasons and often have full-time indoor occupations. These factors greatly limit the ability of the females to get any sun exposure and thus are unlikely to obtain any vitamin D from sunlight.

The lowest deficiency incidence in our study was in the non-diabetic, non-Saudi males (51.79%). Some studies have been conducted on non-diabetic young subjects in other regions of Saudi Arabia and showed a very high incidence of vitamin D deficiency, even higher than our results. However, their results also showed a higher incidence in females than in males [20].

Our results showed a variation in vitamin D deficiency incidence in different age groups. The deficiency was lowest in the 1 to 20-year-old group and was highest in the 21 to 40-year-old group. The incidence of vitamin D deficiency in the above 60-year-old group was less than the incidence in 21 to 40 and 40 to 60 groups. Actually, this result was unexpected because these patients spent most of the time indoors with very limited activity. However, we explained this unexpected result on the basis that these patients may take vitamin D regularly as a prophylactic and therapeutic medication of osteoporosis. Adequate levels of vitamin D maintain bone strength and might help prevent osteoporosis in older and non-ambulatory individuals who have difficulty exercising, postmenopausal women, and individuals on chronic steroid therapy [25].

We collected the patients' 25(OH) vitamin D level, fasting blood glucose (FBG), and HbA1c at the same time as the patients' particular visit to the hospital. Lower vitamin D was associated with higher FBG and HbA1c levels and vice versa. These results confirm the findings described in previous literature [9, 11, 13]. Based on these results, we can suggest a strong effect of vitamin D on the development and progress of diabetes.

There are many studies that recommended the evaluation of vitamin D levels in diabetics as described by Nigil Haroon et al. [15]. The presence of vitamin D deficiency is considered a prediction factor and increases the incidence of diabetes. Moreover, some studies proved that vitamin D supplementation and higher levels of vitamin D may decrease the incidence of diabetes or improve the glycemic control of diabetic patients [14, 18, 23].

Based on our study and other studies done in Saudi Arabia, we can conclude that vitamin D deficiency is a big problem; unfortunately, most of the Saudi population is unaware of this problem. There is a great need to increase the awareness of the population about this condition, the factors affecting vitamin D production, the dietary sources of vitamin D, and to guide them about the optimum time and period of exposure to the sun [26-27]. To overcome this problem, many actions are organized and plans should be implemented. Although there is variation in ultraviolet rays based on regional and seasonal variations, Saudi Arabia has sunny days nearly all year long. Vitamin D should be one of the medications prescribed to diabetic patients based on periodic measuring of vitamin D level in these patients. 

## Conclusions

There is a high incidence of vitamin D deficiency among KFU Health Center patients. The vitamin D level was inversely proportional to the level of fasting glucose and HbA1c. There is an evident role of vitamin D deficiency on glucose tolerance in diabetic patients. Increasing population awareness is essential to overcome the widely prevalent vitamin D deficiency in the population of Saudi Arabia. A program should be planned to encourage exposure to the sun in the proper time and for an adequate period
